# Supplementary material for: Electron microscopy visualization of cell-free mitochondrial DNA–containing extracellular vesicles in human plasma, serum, and saliva
Source: medRxiv. 2025 Oct 17:2025.10.15.25338094. Preprint. [Version 1] doi: 10.1101/2025.10.15.25338094 (PMC12633081; doi:10.1101/2025.10.15.25338094)
Supplement: Supplement 1 — Supplemental Figure S1. Detailed schematic of experimental protocol. (A) Blood and saliva collection and cf-plasma, serum, and saliva preparation. (B) Isolation of extracellular vesicles through size exclusion chromatography using Izon’s SEC protocol, with the isolated pellets fixed and sent to Weill Cornell EM core for analysis. (C) To validate that the pellets sent to Weill had the highest possible amounts of cf-mtDNA, qPCR by lysis was performed of all biofluid samples, pooled SEC fractions, and supernatant. [file media-1.pdf]

Supplemental Figure S1

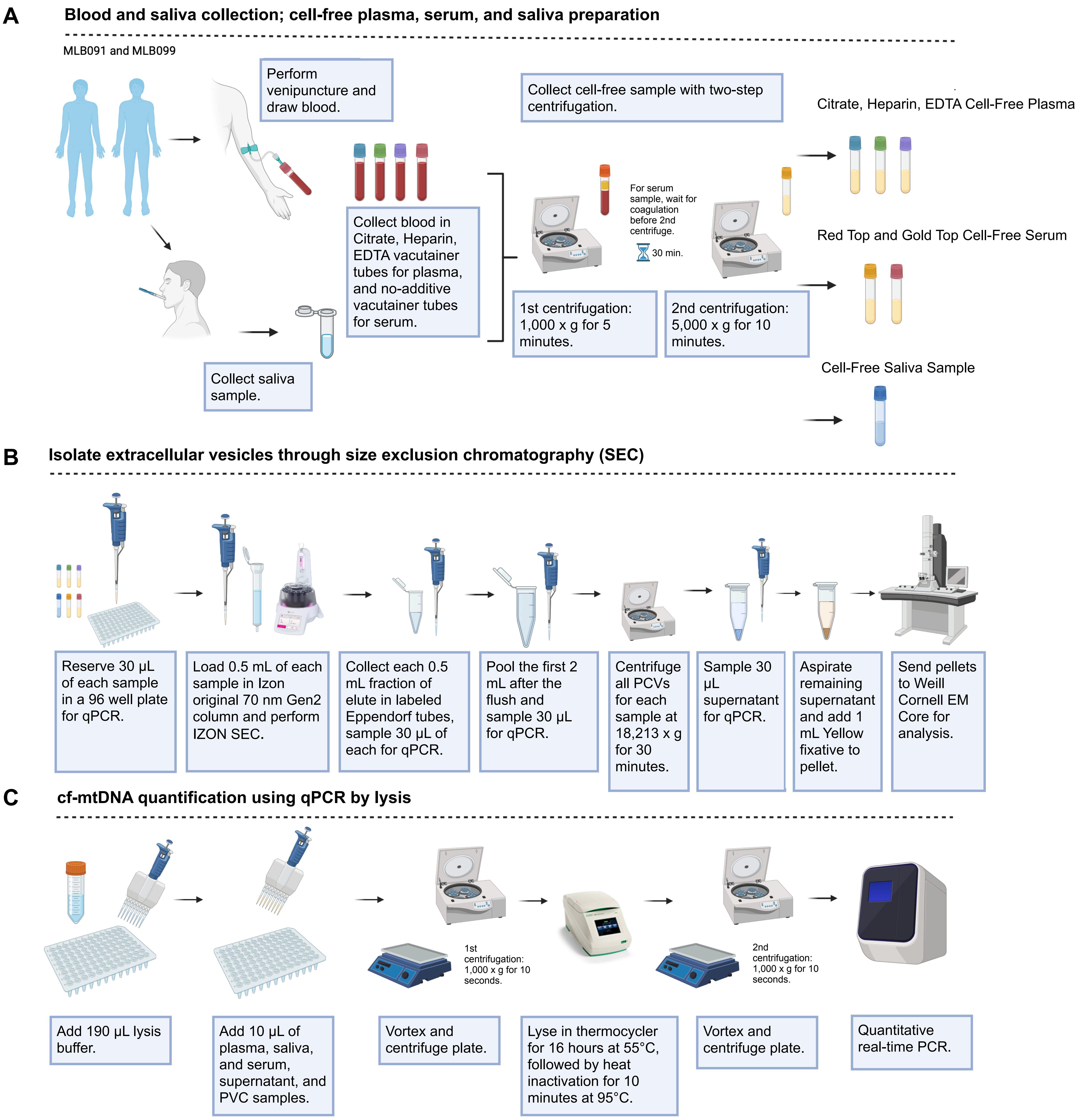

Supplemental Figure S1. Detailed schematic of experimental protocol. (A) Blood and saliva collection and cf-plasma, serum, and saliva preparation. **(B)** Isolation of extracellular vesicles through size exclusion chromatography using Izon's SEC protocol, with the isolated pellets fixed and sent to Weill Cornell EM core for analysis. **(C)** To validate that the pellets sent to Weill had the highest possible amounts of cf-mtDNA, qPCR by lysis was performed of all biofluid samples, pooled SEC fractions, and supernatant.
