# Supplementary material for: Electron microscopy visualization of cell-free mitochondrial DNA–containing extracellular vesicles in human plasma, serum, and saliva
Source: medRxiv. 2025 Oct 17:2025.10.15.25338094. Preprint. [Version 1] doi: 10.1101/2025.10.15.25338094 (PMC12633081; doi:10.1101/2025.10.15.25338094)
Supplement: Supplement 3 [file media-3.docx]

# **ReadMe: Electron Microscopy Image Inventory of Extracellular Vesicles in Human Blood and Saliva**

This repository contains the organized inventory of electron microscopy images used in the paper. The dataset is structured into six main folders, each corresponding to one biofluid type:

1. **Red Top Serum**
2. **Gold Top Serum**
3. **Citrate Plasma**
4. **EDTA Plasma**
5. **Heparin Plasma**
6. **Saliva**

## **File Naming Convention**

Each image file follows the standardized format:

CornellID Order of Imaging ParticipantNumber TubeType_ImageNumber Processing .tif

### **Example:**

22-257 #1 MLB091 Citrate_001 8bit processed.tif

### **Breakdown of Example:**

- **22-257**: Weill Cornell Imaging Core ID assigned to the participant/sample.
- **#1** → Order in which this sample was imaged
- **MLB091** → Participant number.
- **Citrate_001** → Tube type (Citrate plasma in this case) and the sequential image number for that tube.
- **8bit processed.tif** → Image format and processing status (all files are saved as .tif with 8-bit processing applied).

## **Folder Contents**

Each of the six biofluid folders contains only the images corresponding to that biofluid type. Within each folder, filenames carry all necessary metadata (participant ID, sample type, image order, processing status).

## **How to Use the Inventory**

- **Sorting by Biofluid:** Choose the corresponding folder (e.g., "Citrate Plasma") to access images from that biofluid.
- **Locating Participant Data:** Use the **Cornell ID** or **Participant Number** in the filename to locate specific individuals across different biofluids.
- **Comparing Images:** The **Order (#)** value allows differentiation when multiple imaging rounds were performed on the same sample.
- **Tracking Image Processing:** All images are consistently saved in **8bit processed TIFF** format for downstream analyses.

## **Spreadsheet Reference (Image Counts)**

In addition to the folder inventory, a spreadsheet will be provided that details:

- The total image count per biofluid type.
- The total image count per participant across all biofluids.

**Note: Red indicates that the sample was not imaged due to lack of pellet or damaging during processing.**

This spreadsheet is meant to make it easier to:

- Quickly determine how many images exist for a given participant.
- Compare representation of participants across different biofluids.
- Obtain counts without navigating through individual folders.
